# The Impact of Physical Education Attendance and Diet on Bone Mineralization in Adolescents

**DOI:** 10.3390/nu17183016

**Published:** 2025-09-20

**Authors:** Agata Przytula, Joanna Popiolek-Kalisz

**Affiliations:** 1 Department of Clinical Dietetics, Medical University of Lublin, ul. Chodzki 7, 20-093 Lublin, Poland; agata.przytula@umlub.pl; 2 Department of Cardiology, Cardinal Wyszynski Hospital in Lublin, al. Krasnicka 100, 20-718 Lublin, Poland

**Keywords:** bone mineralization, adolescents, physical education, dietary knowledge, bioelectrical impedance analysis

## Abstract

**Background**: Bone mineralization can be influenced by physical activity and dietary factors; however, the relative contributions of these factors are not well defined. Physical activity in adolescents can be implemented with physical education (PE) classes and there is no reported data on their impact on bone mineralization. This study investigates the relationship between PE class attendance, dietary factors, and bone mineralization in adolescents. **Methods**: 57 adolescents (median age 17.00 [16.00–17.00] years) were enrolled in this study. Bone mineralization was assessed with bioelectrical impedance analysis (BIA) and dietary knowledge and selected foods intakes were assessed with a validated questionnaire. **Results**: PE attendance and dietary knowledge were positively associated with bone mineralization in the multivariate models (R^2^ = 0.85, *p* < 0.001 for the best model, and R^2^ = 0.81, *p* < 0.001 for the simplified model), and PE attendance was the dominant positive factor among the modifiable ones (B = 0.20, *p* = 0.02, and B = 0.25, *p* = 0.004, respectively). The impact of calcium sources intake was diminished when controlled for PE class attendance. **Conclusions**: PE attendance is one of the key factors of bone mineralization in adolescents. Our study showed that the role of calcium sources intake was diminished when acknowledging physical activity data, however dietary knowledge remained a significant predictor. Enhancing dietary knowledge and promoting physical activity are key targets for improving bone health.

## 1. Introduction

Lifestyle influences 20–40% of an adult’s peak bone mass [1]. Therefore, optimizing lifestyle factors, which affect bone mass and strength, is crucial to reducing the risk of osteoporosis or low bone mass later in life. Skeletal growth involves a coordinated process of bone deposition and resorption, which continues until epiphyseal fusion, typically at the end of the second decade of life. Bone modeling is sensitive to mechanical loading, highlighting the importance of physical activity throughout the growth period. With the onset of puberty and growth spurts, bone mineral accrual accelerates, and peak bone mass is usually achieved in early adulthood. The concept of peak bone mass includes not only bone mass and density, but also structural properties such as microarchitecture, and geometry that provide bone strength [1].

The prevalence of osteoporosis and fragility fractures is increasing with aging populations worldwide. Elderly individuals with fractures face a high risk of poor outcomes, including prolonged hospital stays and premature mortality. Many age-related changes in musculoskeletal health and function observed with aging, such as the loss of bone mineral density, muscle mass, strength, and function, are largely due to physical inactivity and a sedentary lifestyle. Physical inactivity in adolescence and adult life is strongly associated with osteoporosis in older age [2,3,4], while appropriately targeted exercise interventions can preserve bone and muscle mass, thus extending the functional lifespan [5].

Adequate mineral intake is another critical determinant of skeletal development. Dietary deficiencies during childhood and adolescence may disturb mineral imbalances and increase the risk of metabolic bone diseases [6]. Bone health is closely linked to the intake of nutrients, such as proteins, inorganic salts, vitamins, and more [7]. Calcium plays a key role in bone mineralization and homeostasis, and vitamin D regulates its absorption and utilization [8,9]. Dairy consumption is particularly important in children and adolescents when demands are highest [10]. A balanced diet can prevent bone loss in postmenopausal women, and prevent the occurrence of senile osteoporosis [10].

Bone mineralization assessment in pediatric populations requires methods that are accurate yet safe. Dual-energy X-ray absorptiometry (DEXA) remains the reference method, but its use in children is limited by radiation exposure, cost, and technical challenges [11]. That is why bioelectrical impedance analysis (BIA) emerged as an alternative. BIA is non-invasive, portable, and inexpensive, and in addition to fat mass (FM) and fat-free mass (FFM), it estimates bone mineralization and total body water (TBW) [12,13]. It is widely used in both clinical and non-clinical settings [14]. We chose BIA over DEXA in order to avoid exposing children to radiation, and because subtle bone mineralization changes at a young age might not be detected by a DEXA scan. Moreover, BIA was successfully used for bone mineralization assessment in other studies [15,16].

Physical activity in children can be implemented in multiple ways, and participation in physical education (PE) classes is one of the most structured opportunities. Despite the known relationship between general physical activity and bone health, there are no available studies which analyze the role of PE class participation in bone mineralization. As schools are a central environment for shaping healthy behaviors, it is important to determine if PE attendance can play role in bone development. The aim of this study was to evaluate whether attendance in PE classes and adherence to a balanced diet are significant predictors of bone mineralization in adolescents. We hypothesized that PE class attendance, due to its regularity and intensity, would be a stronger determinant than dietary intake alone.

## 2. Materials and Methods

The students were recruited for this study from schools in the city of Lublin, and smaller towns on a voluntary basis following an invitation sent to schools. Inclusion criteria were as follows: student status, at least 14 years old, informed consent to participate given by the participant, and informed consent for the child’s participation given by the parent or legal guardian in the case of minor participants (<18 years old). Participants with chronic diseases, hormonal disorders, or those taking medications affecting bone metabolism were excluded from this study. Individuals with contraindications for BIA were also excluded, i.e., after amputations, or with large metal implants. Adolescents included in the study were therefore healthy volunteers without chronic metabolic, endocrine, or musculoskeletal disorders, and with no history of conditions or medication affecting bone or mineral metabolism or dietary habits.

Bone mineralization was assessed with BIA with a Tanita MC-780 analyzer (Tanita Corporation, Tokyo, Japan). BIA measurement is based on sending a low-voltage alternating current through the body and measuring the impedance encountered. Since different tissues (e.g., fat and muscle) conduct electricity differently, BIA can estimate their proportions. The measurement was performed by qualified dietician. To minimize variability of BIA results, participants were instructed to follow standard pre-test recommendations: refrain from vigorous physical activity for at least 12 h before the measurement, avoid food and caloric beverages for at least 3–4 h before the test, abstain from caffeine and energy drinks on the day of measurement, ensure normal hydration but avoid excessive fluid intake immediately before the test, and empty the bladder within 30 min before measurement. All measurements were performed in the morning, in light clothing, and in a standing position according to the device manufacturer’s guidelines.

Dietary intake data and dietary knowledge were assessed with the Dietary Habits and Nutrition Beliefs Questionnaire for adolescents and adults [17]. The questionnaire is composed of 4 parts, and part B was utilized in the study to examine dietary habits, while part C was used to assess dietary knowledge level. The questionnaire is a validated qualitative tool. It includes questions about dietary habits and a list of 33 food groups, allowing for the characterization of the respondent’s usual diet over the past 12 months. Respondents can choose from 6 frequency categories for food consumption: never, 1–3 times a month, once a week, several times a week, once a day, and several times a day. The data obtained are in a qualitative format and then converted into semi-quantitative data that reflect daily food consumption frequency (times/day). Based on the frequency of health-promoting foods, the pro-healthy diet index (pHDI) is calculated, and for unhealthy foods, the non-healthy diet index (nHDI) is determined. From these indices, the diet quality index (DQI) is calculated as an overall diet quality indicator. All these indices are expressed in points, and the calculations were based on the instruction developed by authors of the questionnaire. The other part of the questionnaire contains 25 statements about food and nutrition, which enable the assessment of the respondents’ level of dietary knowledge. The respondents were asked to assess true, false or I do not know regarding these statements. The dietary knowledge score is based on the number of right answers and is expressed in points. Based on the collected data, respondents can be ranked in terms of food consumption and/or their characteristic food consumption patterns can be identified.

Physical activity classes attendance was assessed by questionnaire in which participants were asked if they participate in PE classes. They were also asked what their self-assessed level of physical activity at school (apart from PE) and in their free time was, as they could choose the options low/lack of activity, moderate, or high.

The normality of distribution was assessed with a Shapiro–Wilk test. The data were presented as mean ± standard deviation (SD) for normal-distributed data, and median [interquartile range (IQR)] for non-normal distribution, or number of cases (percentage), depending on the data type. The comparison between two subgroups was performed with the Welch *t*-test or Mann–Whitney test for continuous variables, and the chi-square test for categorical variables. The relationship between available parameters and bone mineralization was investigated with the regression model adjusted for diet quality indices, dietary intake data, and dietary knowledge. A *p*-value below 0.05 was considered significant. Statistical analyses were performed using STATA/BE 18.0 software (StataCorp LCC, College Station, TX, USA) and RStudio (ver. 4.4.2).

The study was approved by the local Bioethics Committee of Medical University of Lublin Decision number KE-0254/28/01/2024 of 25 January 2024. The participants and their parents (legal guardians) signed a consent form for participation in the study.

## 3. Results

### 3.1. Basic Characteristics of the Study Group

**Figure 1:**
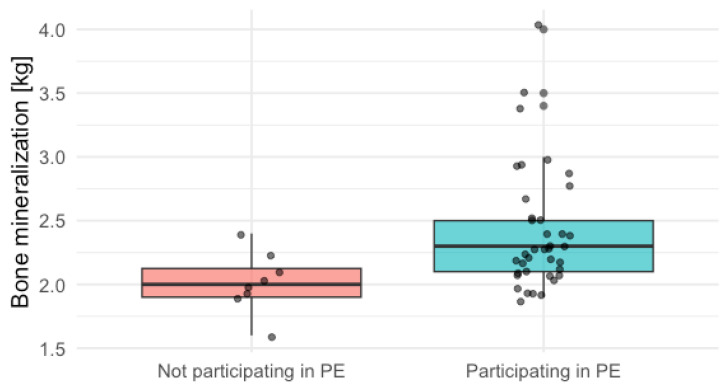
Box-plot comparing bone mineralization in adolescents who participated in PE and did not.

A total of 57 adolescents (median age 17.00 [16.00–17.00] years) were enrolled in this study. Complete information about PE class attendance, dietary data, and bone mineralization was available for 47 participants. Among them, 19.15% were not participating in PE classes. The comparison between children who participated in PE classes showed that the subgroup participating in PE had significantly better bone mineralization than the ones who did not participate (2.30 [2.10–2.50] vs. 2.00 [1.90–2.12], *p* = 0.01). It is interesting that there were no significant differences in dietary intake, quality, or knowledge indices. Detailed results are presented in Table 1 and the visual presentation in a box-plot in Figure 1.

### 3.2. Bone Mineralization and Physical Activity

The detailed analysis of the available data on physical activity at school and free time was performed with univariate analyses between PE class attendance, the volume of additional voluntary physical activity at school and in free time. The results indicated that PE class attendance significantly impacted bone mineralization (B = 0.40, *p* = 0.03). The relationship was not significant for other aspects of physical activity. Detailed results of univariable regressions are presented in Table 2.

In a multivariable model, the impact of PE class attendance was still significant when controlled for age, sex, and BMI, which was presented in Table 3. The overall model was still significant with R^2^ of 0.74 and *p* < 0.001.

### 3.3. Bone Mineralization and Diet

The impact of selected products’ intake and diet quality was initially investigated with univariate regression models. The univariate analyses indicated significant impact on bone mineralization of plain and wholegrain products (B = 0.67, *p* = 0.02 and B = 0.81, *p* < 0.001, respectively), fermented milk products (B = 0.43, *p* = 0.04), legumes (B = 1.83, *p* < 0.001), canned meat (B = 3.13, *p* < 0.001), and dietary knowledge level (B = 0.04, *p* < 0.001). When controlled for age, sex, and BMI the significant relationship was presents for nuts (B = −0.66, *p* = 0.04), potatoes (B = −0.43, *p* = 0.01), and canned meat (B = 1.21, *p* = 0.02). There was no significant relationship observed for products typically rich in calcium. The detailed results are presented in Table 4.

### 3.4. Combined Multivariable Models

Further multivariable analyses were performed in order to find the best model for bone mineralization prediction. When focused only on calcium sources, the best model was composed of milk-derived products, eggs, nuts, and vegetables (R^2^ = 0.79, *p* < 0.001), however only fermented milk products, nuts, and vegetables were the significant factors.

When focused on PE attendance, diet quality, and knowledge, the best model to predict bone mineralization was based on PE attendance, DQI, and dietary knowledge (*p* < 0.001, R^2^ = 0.34). When adjusted for age, sex, and BMI the model was even better (R^2^ = 0.79, *p* < 0.001).

When analyzing selected products’ intake, the best model was based on PE class attendance, white bread, wholegrain bread, plain pasta, wholegrain pasta, fast food, nuts, fermented milk products, white cottage cheese, red meat, eggs, potatoes, fruits, ready soups, canned meat, fruit juices, hot drinks, sweetened drinks, and butter (R^2^ = 0.77, *p* < 0.001).

When controlled for BMI, sex, and age, the best model included PE class attendance, plain pasta, wholegrain pasta, nuts, red meat, eggs, potatoes, fruits, ready soups, canned meat, fruit juices, sweetened drinks, male sex, and BMI (R^2^ = 0.91, *p* < 0.001).

When choosing between dietary quality indexes and calcium sources, the best model also included dietary knowledge as important factor (B = 0.03, *p* = 0.01). Compared to models which acknowledged only dietary data, addition of PE class attendance improved the predictability of the model (R^2^ = 0.85, *p* < 0.001). When the model included only significant predictors it retained significant prediction (R^2^ = 0.81, *p* < 0.001). It was based on PE class attendance, BMI, male sex, nuts, vegetables and dietary knowledge. The details of all these models are consecutively presented in Table 5.

## 4. Discussion

Our study demonstrated that participation in PE classes was significantly associated with improved bone mineralization in adolescents. This finding highlights the unique contribution of structured, school-based physical activity. Among various lifestyle components, PE class attendance emerged as the main modifiable predictor. This finding addressed an important gap in the literature, as most of the available evidence linking diet and physical activity to bone mineral density and content in children has been extrapolated from adult studies [18]. Our results highlight the role of the school environment in shaping future health in adolescents and emphasize the unique contribution of structured school-based physical activity, as it surpasses the impact of self-reported free-time physical activity, which did not show a statistically significant association. This is the first study which demonstrated this association. No significant differences were observed in terms of dietary intake, quality, or nutritional knowledge indicators among children who participated in PE classes and the ones who did not participate, which shows that PE class attendance was not associated with dietary attitudes or knowledge.

In multivariable prediction models, PE class attendance remained a significant predictor of bone mineralization even when controlling for age, sex, and BMI, confirming its robustness. Alongside PE class attendance, dietary knowledge, and selected food items (nuts, vegetables) also emerged as contributors to bone health. These findings align with the HELENA study, which demonstrated that both physical activity and fitness performance are critical determinants of bone mass in Spanish adolescents [19]. However, the authors of that study did not analyze the source of physical activity. These results underscore that the relationship between exercise and bone mineralization is complex, involving not only time spent in activity but also its type, intensity, and the resulting adaptations in muscle mass and mechanical loading. Similarly, the Iowa Bone Development Study demonstrated that sustained moderate-to-vigorous physical activity from early childhood to late adolescence were strong predictors of bone mass and geometry [20]. Together with our results, this evidence suggests that school-based structured physical activity offers a unique opportunity to influence bone health during adolescence. Moreover, the longitudinal HR-pQCT study by Gabel et al. demonstrated that moderate-to-vigorous physical activity predicts favorable changes in bone geometry in adolescents, while sedentary time was associated with adverse effects [21]. It is worth noting that the role of physical activity in bone mineralization is not limited to adolescents as it was also reported, e.g., for young men [16].

Self-reported voluntary activity, either at school outside PE classes or during free time, was not significantly related to bone mineralization in our study. This may reflect the subjective nature of self-reports, but it also indicates that irregular or low-intensity activity may not be sufficient to provide mechanical loading required for skeletal adaptation. Evidence from interventional studies demonstrates that only weight-bearing physical activities, such as jumping, may contribute to increased bone mineral density in children [22]. Moreover, the study by T. Lloyd et al. indicates that exercise is the dominant lifestyle factor influencing bone strength in adolescent and young women, while daily calcium intake ranging from 500 to 1900 mg/day showed no significant association which bone mass gain [23]. Other studies also support a link between exercise and better bone development [24]. In agreement with our findings, these results reinforce the hypothesis that mechanical stress from structured exercise, rather than dietary calcium alone, is the key driver of bone mineralization during adolescence. This is also in line with the updated World Health Organization guidelines, which recommend that children and adolescents accumulate an average of 60 min per day of moderate-to-vigorous physical activity and include regular muscle- and bone-strengthening exercises [25].

In contrast, dietary intake of typical calcium-rich products did not remain significant when PE class attendance was included in the model, while nutritional knowledge demonstrated an independent positive association with bone mineralization. This suggests that knowledge-driven dietary choices may play a complementary role to physical activity. Although several food products (whole grains, fermented dairy products, legumes, and canned meats) demonstrated associations with bone mineralization in univariate analyses, these associations were not retained in multivariate models. That is why our findings suggest that the dominant driver of bone mineralization was PE class attendance, whereas diet had only a secondary or modulatory role. This aligns with evidence that a balanced diet rich in protein, calcium, vitamin D [26], fruits and vegetables [27], or general adherence to a Mediterranean diet contribute to bone health [28], but mechanical loading remains indispensable for bone mineralization. It is worth noting that our results also indicate that adolescents with higher nutritional knowledge may adopt healthier behaviors, which may indirectly benefit bone health. Available data suggests that individuals with higher nutritional literacy make better dietary choices [29], which may potentially include proper calcium and protein intake.

The absence of significant effects from calcium-rich foods when controlling for PE class attendance suggests that mechanical loading through exercise may play a dominant role in bone accrual during adolescence. This does not mean that dietary factors are unimportant, as adequate calcium and protein intake, together with vitamin D, remain essential for optimal skeletal development in Polish children as well [22,30]. Observational studies on Polish boys also confirm that bone mineralization is highly dependent on moderate plus vigorous activity, sit time, along with the intake of dairy products, calcium, protein, vitamin D, and phosphorus from their diet [31]. Moreover, age, visceral fat mass, and vitamin D intake were identified as predictors of bone strength in young adults [32]. However, our results suggest that in the short term, the effects of diet may be masked by the much stronger influence of physical activity. Interestingly, our models indicated a negative association between vegetable consumption and bone mineralization. This may be explained by the heterogeneity of vegetables in terms of calcium content and bioavailability. While cruciferous vegetables (e.g., broccoli) contain bioavailable calcium and may contribute positively to bone health, other vegetables contain compounds that reduce absorption [33]. Our study did not differentiate between vegetable subtypes, which may explain the observed negative association, and it should be interpreted with caution. Further research on detailed types of vegetables is needed to investigate that observation. On the other hand, in the course of looking for the simplest model of bone mineralization, which included all lifestyle aspects, vegetable consumption was not significant factor, but only a modificatory aspect.

Finally, our results confirm that bone mineralization outcomes are influenced by a combination of factors including sex, BMI, and lifestyle, but that PE class attendance consistently remained the main determinant. While univariate analyses identified nuts, potatoes, and canned meats as significant dietary predictors, in multivariate models, only nuts and vegetables remained significant. This reinforces the concept that both physical activity and diet interact in shaping skeletal outcomes, but structured exercise has a dominant role. The inclusion of nutritional knowledge in the best-fitting models further highlights the importance of integrating educational strategies with physical activity promotion. Adolescents with higher knowledge may potentially make conscious dietary choices including calcium-rich products and protein, as well as avoid other risk factors, which collectively contribute to bone health.

Although the study has benefits, which include the non-invasive assessment of bone mineralization in adolescents and the use of validated tools along with important co-variates, this study has its limitations due to its cross-sectional design, and it being based on self-reported data. Moreover, the research group size was limited, so further research on larger groups is needed to confirm these preliminary observations.

## 5. Conclusions

Attendance in PE classes is one of the key factors of bone mineralization in adolescents. Our study showed that the role of calcium source intake was diminished when acknowledging physical activity data, however dietary knowledge remained a significant predictor. Moreover, this study showed the potential of using BIA as a non-invasive tool for bone mineralization assessments in adolescents and highlighted the role of participation in PE classes for proper bone development.

## Figures and Tables

**Table 1:** Basic characteristics of the study group and comparison between adolescents who participate in PE and those who do not participate.

Parameter	Overall (n = 47)	Participating in PE (n = 38)	Not Participating in PE (n = 9)	*p*
Men [%]	14%	18%	13%	0.71
BMI [kg/m^2^]	20.89 [19.13, 23.11]	21.20 [19.79, 23.27]	18.39 [17.91, 20.96]	0.070
Age [years]	17.00 [16.00, 17.00]	17.00 [15.25, 17.00]	16.50 [16.00, 17.00]	0.831
Bone mineralization [kg]	2.20 [2.10, 2.48]	2.30 [2.10, 2.50]	2.00 [1.90, 2.12]	0.009
Selected products intake [portion/day]				
White bread	0.50 [0.32, 1.00]	0.50 [0.23, 1.00]	2.00 [0.50, 2.00]	0.104
Wholegrain bread	0.50 [0.06, 0.50]	0.50 [0.08, 0.50]	0.50 [0.06, 0.50]	0.899
Plain pasta	0.50 [0.14, 0.50]	0.50 [0.14, 0.50]	0.50 [0.06, 0.50]	0.435
Wholegrain pasta	0.14 [0.06, 0.50]	0.14 [0.06, 0.50]	0.06 [0.06, 0.50]	0.810
Fast food	0.06 [0.06, 0.14]	0.06 [0.06, 0.14]	0.06 [0.06, 0.06]	0.169
Nuts	0.06 [0.03, 0.06]	0.06 [0.06, 0.06]	0.06 [0.00, 0.06]	0.197
Fried meals	0.50 [0.14, 0.50]	0.50 [0.14, 0.50]	0.14 [0.14, 0.50]	0.445
Butter	0.50 [0.14, 1.00]	0.50 [0.14, 1.00]	0.50 [0.50, 2.00]	0.440
Lard	0.00 [0.00, 0.00]	0.00 [0.00, 0.00]	0.00 [0.00, 0.00]	0.985
Margarine	0.06 [0.00, 0.14]	0.06 [0.00, 0.14]	0.00 [0.00, 0.14]	0.511
Milk	0.50 [0.14, 0.50]	0.50 [0.14, 0.50]	0.50 [0.14, 1.00]	1.000
Fermented milk products	0.14 [0.06, 0.50]	0.14 [0.06, 0.50]	0.06 [0.06, 0.50]	0.492
White cottage cheese	0.06 [0.06, 0.14]	0.06 [0.06, 0.14]	0.06 [0.06, 0.06]	0.438
Yellow cheese	0.50 [0.14, 0.50]	0.50 [0.14, 0.50]	0.14 [0.14, 0.50]	0.569
Processed meat	0.50 [0.14, 0.50]	0.50 [0.14, 0.50]	0.50 [0.50, 1.00]	0.097
Red meat	0.06 [0.03, 0.14]	0.06 [0.01, 0.14]	0.06 [0.06, 0.06]	0.536
White meat	0.50 [0.14, 0.50]	0.50 [0.14, 0.50]	0.14 [0.14, 0.50]	0.150
Fish	0.06 [0.06, 0.14]	0.06 [0.06, 0.06]	0.06 [0.06, 0.14]	0.610
Eggs	0.14 [0.06, 0.50]	0.14 [0.08, 0.50]	0.14 [0.06, 0.50]	0.724
Legumes	0.06 [0.00, 0.06]	0.06 [0.00, 0.06]	0.06 [0.00, 0.06]	0.456
Potatoes	0.50 [0.14, 0.50]	0.50 [0.14, 0.50]	0.50 [0.14, 0.50]	0.817
Fruits	0.50 [0.50, 1.00]	0.50 [0.50, 1.00]	0.50 [0.14, 0.50]	0.332
Vegetables	0.50 [0.50, 2.00]	0.50 [0.50, 2.00]	1.00 [0.50, 2.00]	0.648
Sweets	0.50 [0.50, 1.00]	0.50 [0.50, 1.00]	0.50 [0.50, 1.00]	0.850
Ready soups	0.06 [0.00, 0.06]	0.06 [0.00, 0.06]	0.06 [0.00, 0.06]	0.751
Canned meat	0.00 [0.00, 0.00]	0.00 [0.00, 0.00]	0.00 [0.00, 0.00]	0.540
Canned vegetables	0.06 [0.00, 0.14]	0.06 [0.00, 0.14]	0.06 [0.06, 0.14]	0.198
Fruit juices	0.14 [0.10, 0.50]	0.32 [0.14, 0.50]	0.14 [0.06, 0.50]	0.899
Vegetable juices	0.06 [0.00, 0.06]	0.06 [0.00, 0.06]	0.00 [0.00, 0.00]	0.088
Hot drinks	0.50 [0.06, 1.50]	0.50 [0.06, 1.00]	2.00 [0.50, 2.00]	0.062
Sweetened drink	0.06 [0.06, 0.14]	0.06 [0.06, 0.14]	0.06 [0.06, 0.14]	0.876
Energy drinks	0.06 [0.00, 0.14]	0.00 [0.00, 0.12]	0.06 [0.00, 0.50]	0.324
Water	2.00 [2.00, 2.00]	2.00 [2.00, 2.00]	2.00 [2.00, 2.00]	0.798
Alcohol	0.00 [0.00, 0.06]	0.00 [0.00, 0.06]	0.00 [0.00, 0.00]	0.465
Diet quality and knowledge [points]				
pHDI	19.00 [11.40, 25.25]	19.20 [11.60, 26.00]	18.70 [10.10, 23.10]	0.579
nHDI	16.21 [12.00, 20.57]	16.18 [12.36, 19.93]	17.79 [9.50, 31.29]	0.561
DQI	2.88 ± 12.60	3.97 ± 10.90	−1.70 ± 18.27	0.394
Dietary knowledge	10.00 [8.00, 13.00]	10.00 [8.00, 13.00]	11.00 [8.00, 16.00]	0.870

BMI—body mass index, DQI—diet quality index, nHDI—non-healthy diet index, PE—physical education, pHDI—pro-healthy diet index.

**Table 2:** Univariable regressions between physical activity and bone mineralization.

Predictor	B	*p*	R^2^
PE attendance	0.40	0.03	0.11
Additional physical activity at school	−0.11	0.35	0.02
Physical activity at free time	0.17	0.06	0.08

PE—physical education.

**Table 3:** Multivariable model for impact of PE on bone mineralization.

Predictor	B	*p*	R^2^	*p* for Model
PE attendance	0.22353	0.0242	0.74	<0.001
Male sex	0.70967	<0.001		
Age	0.02107	0.5360		
BMI	0.05635	<0.001		

PE—physical education.

**Table 4:** Univariate regression of selected food groups intake impact on bone mineralization.

Predictor	B	*p*	Age, Sex and BMI—Adjusted	
			B	*p*
White bread	−0.16	0.17	−0.04	0.54
Wholegrain bread	0.22	0.15	−0.16	0.09
Plain pasta	0.67	0.02	0.26	0.12
Wholegrain pasta	0.81	<0.001	0.03	0.87
Fast food	0.48	0.39	0.53	0.09
Nuts	0.26	0.66	−0.66	0.04
Fried meals	0.07	0.69	−0.11	0.21
Butter	0.04	0.73	0.01	0.86
Lard	0.03	0.95	0.26	0.23
Margarine	0.40	0.21	0.02	0.90
Milk	−0.01	0.97	−0.09	0.20
Fermented milk products	0.43	0.04	−0.03	0.80
White cottage cheese	0.33	0.15	0.17	0.21
Yellow cheese	0.23	0.14	0.11	0.20
Processed meat	−0.14	0.23	−0.03	0.65
Red meat	0.42	0.19	−0.19	0.28
White meat	0.31	0.14	−0.07	0.55
Fish	0.75	0.29	0.03	0.94
Eggs	0.25	0.21	−0.14	0.21
Legumes	1.83	<0.001	0.40	0.25
Potatoes	0.12	0.71	−0.43	0.01
Fruits	0.01	0.93	−0.05	0.41
Vegetables	−0.04	0.66	−0.08	0.14
Sweets	−0.15	0.21	0.01	0.88
Ready soups	−0.02	0.95	0.16	0.42
Canned meat	3.13	<0.001	1.21	0.02
Canned vegetables	0.38	0.08	0.15	0.22
Fruit juices	0.05	0.75	0.00	0.98
Vegetable juices	0.41	0.12	−0.21	0.20
Hot drinks	−0.14	0.13	−0.06	0.25
Sweetened drink	0.19	0.48	0.02	0.90
Energy drinks	−0.26	0.35	−0.17	0.30
Water	0.12	0.34	0.05	0.48
Alcohol	0.83	0.66	0.94	0.41
pHDI	0.01	0.16	0.00	0.20
nHDI	0.00	0.99	0.00	0.95
DQI	0.01	0.19	0.00	0.23
Dietary knowledge	0.04	<0.001	0.01	0.11

BMI—body mass index, DQI—diet quality index, nHDI—non-healthy diet index, pHDI—pro-healthy diet index.

**Table 5:** Multivariate regression models for bone mineralization prediction.

Predictor	B	*p*	R^2^	*p*
Milk	−0.11071	0.12	0.79	<0.001
Fermented milk products	0.358265	0.04		
Yellow cheese	0.246495	0.01		
Eggs	−0.26262	0.09		
Nuts	−0.75367	0.02		
Vegetables	−0.11638	0.03		
Butter	−0.07461	0.20		
BMI	0.076923	<0.001		
Age	0.044405	0.19		
Male sex	0.606996	<0.001		
PE attendance	0.34	0.03	0.34	<0.001
DQI	0.01	0.16		
Dietary knowledge	0.07	<0.001		
PE attendance	0.23	0.01	0.79	<0.001
Dietary knowledge	0.03	0.01		
Male sex	0.64	<0.001		
BMI	0.05	<0.001		
PE attendance	0.42	0.001	0.77	<0.001
White bread	0.09	0.26		
Wholegrain bread	0.30	0.02		
Plain pasta	0.49	0.02		
Wholegrain pasta	1.19	<0.001		
Fast food	0.77	0.049		
Nuts	−1.07	0.01		
Fermented milk products	0.60	0.001		
White cottage cheese	−0.37	0.04		
Red meat	0.69	0.01		
Eggs	−0.62	0.004		
Potatoes	−0.66	0.004		
Fruits	−0.18	0.04		
Ready soups	−0.96	0.001		
Canned meat	3.74	<0.001		
Fruit juices	0.25	0.03		
Hot drinks	0.11	0.05		
Sweetened drink	−0.49	0.01		
Butter	0.08	0.28		
PE attendance	0.27	<0.001	0.91	<0.001
Plain pasta	0.23	0.05		
Wholegrain pasta	0.43	<0.001		
Nuts	−1.05	<0.001		
Red meat	0.22	0.10		
Eggs	−0.17	0.04		
Potatoes	−0.67	<0.001		
Fruits	−0.06	0.12		
Ready soups	−0.42	0.01		
Canned meat	2.10	<0.001		
Fruit juices	0.15	0.03		
Sweetened drink	−0.27	0.01		
Male sex	0.60	<0.001		
BMI	0.05	<0.001		
PE attendance	0.2	0.02	0.85	<0.001
BMI	0.06	<0 001		
Male sex	0.54	<0 001		
Milk	−0.09	0.13		
Fermented milk products	0.26	0.07		
Yellow cheese	0.16	0.06		
Eggs	−0.18	0.16		
Nuts	−0.81	0.004		
Vegetables	−0.1	0.045		
Butter	−0.07	0.23		
Legumes	0.41	0.17		
Dietary knowledge	0.03	0.01		
PE attendance	0.25	0.004	0.81	<0.001
BMI	0.06	<0.001		
Male sex	0.65	<0.001		
Nuts	−0.69	0.01		
Dietary knowledge	0.03	0.01		

BMI—body mass index, DQI—diet quality index, PE—physical education.

## Data Availability

The original contributions presented in this study are included in the article. Further inquiries can be directed to the corresponding author.
